# Good weather for a ride (or not?): how weather conditions impact road accidents — a case study from Wielkopolska (Poland)

**DOI:** 10.1007/s00484-023-02592-3

**Published:** 2023-12-07

**Authors:** Iwona Pińskwar, Adam Choryński, Dariusz Graczyk

**Affiliations:** 1 Department of Land Improvement, Environmental Development and Spatial Management, Faculty of Environmental Engineering and Mechanical Engineering, Poznan University of Life Sciences, Piątkowska 94E, 60- 649 Poznań, Poland; 2 Meteorology Laboratory, Department of Construction and Geoengineering, Faculty of Environmental Engineering and Mechanical Engineering, Poznan University of Life Sciences, Piątkowska 94, 60-649 Poznań, Poland

**Keywords:** Road accidents, Weather-related risk, Extreme weather, Poland, Wielkopolska

## Abstract

This study offers a likely assessment of extreme meteorological events’ impact on human perceptivity, frame of mind or even health during driving which might have had a consequence as a car accident. Research covered an analysis of car accidents during period 2010–2019 in the Wielkopolska (Poland) and four indices like maximum daily temperature, maximum value of humidex, difference between maximum temperatures observed from day to day and also difference between mean atmospheric pressure at the sea level observed from day to day. A distributed lag nonlinear model (DLNM) approach was used to obtain the relationship between these indices and car accidents. Our finding evidence that the “good weather for a ride” conditions are actually generating an increased risk of accidents. For indices related to high temperature, i.e., maximum temperature and humidex, it was possible to identify the critical values by which the risks of car accidents were the highest.

## Introduction

The observed global increase in air temperature (Seneviratne et al. 2021) is conducive to the appearance of more extreme meteorological events, sometimes more violent. Impact of climate change on several sectors like agriculture, infrastructure, energy, industry, and productivity in general as well as on human beings, health, and life is a subject of numerous studies. Among them consequences of extreme meteorological events, like heat waves (Robine et al. 2007; Barriopedro et al. 2011; Gabriel and Endlicher 2011; Urban et al. 2014; Graczyk et al. 2019; Kuchcik 2021), droughts (Bachmair et al. 2017; Van Lanen et al. 2016; Bastos et al. 2020; Gu et al. 2020; Pińskwar et al. 2020; Yang et al. 2023), or flooding (cf. Kron et al. 2019; Kundzewicz et al 2020; Sieg and Thieken 2022; Lu et al. 2023; Oke et al. 2023), are the most presented around the world.

Disasters like hurricanes, floods, or extreme heat waves often receive more public attention than the gradual effects of climate change, but the latter also cause significant harm to human health, especially mental health, such as psychological distress, anxiety, and depression (Trombley et al. 2017). Broader research revealed a wider spectrum of health risks due to the social, demographic, and economic disruptions of climate change (McMichael et al. 2006). Many studies analyzed high air temperatures concerning in increased mortality among at-risk populations, such as elderly people or people with cardiovascular diseases (Kenney et al. 2014; Yin and Wang 2017; Rodrigues et al. 2020; 2021). This is not only a problem of large agglomerations; also, people living in small towns and villages are under increased risk of mortality during heat waves (Graczyk et al. 2022) and other extreme weather events resulting in large losses (Choryński et al. 2023). The high air temperature has also influence on the psychotic exacerbation of symptoms for many specific mental and behavioral disorders (Semenza et al. 1999; McMichael et al. 2006; Wang et al. 2014) or even on increasing risk of suicide (Kim et al. 2019).

Extreme temperatures are not the only risk factor. Sudden changes in temperatures from low to high and vice versa in a very short time may cause many health problems. Abrupt changes in day-to-day temperatures are linked with higher mortality rates in weather-suicide associations, increasing in hospitalization and higher risk of influenza outbreaks (Holopainen et al. 2014; Dixon and Kalkstein 2018, Zhou et al. 2020; Ombadi and Risser 2022). Another weather-related issue which can influence health is changes in atmospheric pressure (Hiltunen et al. 2012). Also, allergic diseases are a major public health problem globally and are on the rise due to increase in air temperature (Asher et al 2020; World Health Organization 2023; Ziska et al. 2019).

Besides speeding and poor road conditions, all of the above-mentioned elements may have an impact on road injuries. Among the road users, there are both people from risk groups as well as healthy people. Each traffic participant is more or less exposed to the adverse effects of weather conditions. Depending on the impact of extreme weather conditions, unfavorable symptoms among drivers may increase and results in more road accidents. Among the factors one can mention distress, anxiety, depression, lack of sleep, distractions, driver error, fatigue or sleepiness, or even worsening of eyesight through tearing and distraction by sneezing caused by allergens. Additionally, some medicants might increase the risk of weather-related illness, like psychotropic medications during heat waves (Christenson et al. 2013), especially when depression is becoming an increasingly common disease.

Basagaña et al. (2015) reported significantly increase by 2.9% in risk of crashes during heat wave days in Catalonia, and even up to 7.7%, when driver performance–associated factors, like distractions, driver error, fatigue, or sleepiness were taken into account. The estimated risk of crashes with such factors significantly increased by 1.1% for each 1°C increase in maximum temperature. In study for Spain (Basagaña and de la Peña-Ramirez 2023), increase in the risk of crashes with associated factors was even higher up to 23%. He et al. (2023) in their globally research stated that two indices, namely age-standardized mortality rates (ASMR) and age-standardized disability-adjusted life years rates (ASDR) of the road injury attributable to high temperature have decreased, but the absolute death and disability-adjusted life years (DALYs) are on the rise.

According to Vos et al. (2020) road injuries are ranked first as injury causes of disability-adjusted life-years in two groups aged 10–24 and 25–49 years. Polish roads are identified as one of the most dangerous in Europe (ETSC 2022). There were above 110 road deaths per million inhabitants recorded in 2011 (the highest number in Europe: 4189) and above 59 in 2021 (2245; the sixth highest) by EU27 average of 46. Despite a growing number of new sections of expressways and highways, the number of car accidents for last years has been decreasing, but still is very high when comparing to other developed countries. In turn, the road risk, i.e., deaths per vehicle/km travelled, which was estimated for the 25 countries (for Poland for 2017–2019), was the fourth highest for Poland: 11.41 deaths by the EU19 2018–2020 mean 5.12 (ETSC 2022). In reference to information provided by the Police, in Poland drivers very often do not adapt to the road conditions, also the number of drivers under the influence of alcohol is high: in 2021 there were 58,085 cases of detected offences in Poland and 4853 in Wielkopolska (Statistics of Police 2022). Also, the technical condition of vehicles is often insufficient. Only 10% of passenger cars registered in Poland (as at the end of 2021, GUS, 2023), are aged below 6 years. Above 75% of vehicles are 12 years and older. Among them, the group from 21 to 30 years accounted for 23.9% and 17.4% above 30 years of all passenger cars registered in the Central Vehicle Register. Moreover, nearly 75% of trucks are 12 years and older. According to statistics of the Traffic Bureau of theCentral Police Headquarters (2011–2023), most of the accidents in the period 2010–2022 took place during the summer months (June–August) and also in October. On the one hand, this is due to increased traffic during the holiday season, on the other because of the so-called “good weather conditions”, at which much higher speeds are developed and at which the highest number of accidents occur. Furthermore, the number of road users like pedestrians, bicyclists, and motorcyclists is higher as the temperature increases. Hjelkrem and Ryeng (2016) analyzed how precipitation, light conditions and surface conditions affect the drivers’ risk perception, when the normal condition (i.e., “clear”, “dry”, and “daylight”) was defined as the reference situation. Their results showed that both car and truck drivers perceive the highest risk when driving snow-covered roads. For car drivers a snow-covered road in combination with moderate rain or light snow contributed to the perception of increased risk, for truck drivers precipitation did not affect this perception.

Driving behavior is affected by adverse conditions, accident rates are changing as well. Rain and snow influence speed reduction at most. The higher intensity of precipitation, the slower speed (Agarwal et al. 2005; Billot et al. 2009). Mohamed et al. (2013) conducted research on the severity of pedestrian–vehicle crashes in NY, USA, and Montreal, Canada, and they also pointed out that bad weather (cloudy, rainy and snowy) reduces the probability of a fatal crash, and the possible reason is that drivers travel more cautiously. Kim et al. (2010) showed that such types of weather (rain, snow, fog, and smog) much decreased the probability of fatal injuries but increased the probability of minor injuries.

Leard and Roth (2015) estimated the impact of weather on traffic accidents. Their results indicate that fatalities increase with snowfall, decrease with rainfall and also increase with temperature. Authors pointed out that many of these crashes are due to interactions with pedestrians. Based on results, they estimated a probable number of fatalities under a climate change scenario A1B, resulting in 4°C of warming by the end of the century: the shift from snow to rain may result in saving people’s life, but temperature increases will surpass these positive effects, causing more additional fatalities. Authors also linked these fatalities with psychological effects of heat or weather associated changes in aggression.

In that sense “good weather” means that there is no rain, snow, fog, and strong wind and is perceived by drivers as not requiring much vigilance and attention. Meanwhile what cannot be seen, but is felt by the human body, affects the behavior on the road.

Most of studies of the impact of extreme meteorological events on road accidents presented influence of hot or cold temperature (Basagaña et al. 2015; Liu et al. 2017; Wu et al. 2018; Park et al. 2021; Basagaña and de la Peña-Ramirez 2023), some of them on extreme precipitation (Liu et al. 2017; Jaroszweski and McNamara 2014, Keay and Simmonds 2005), snowfall (Eisenberg and Warner 2005; Abohassan et al. 2022), or seasonal variations in road injuries (Gill and Goldacre 2009). The strong wind, low visibility, and temperature were considered in the context of delays caused by a road accident (Su et al. 2023). Abe et al. (2008) studied the impact of weather conditions (barometric pressure, ambient temperature, relative humidity, and rainfall) on the occurrence of trauma among patients transported to hospitals. Forty percent of all trauma patients experienced car collisions in Tokyo. Very few researches present also the impact of other indices of extreme weather on road accidents. This study offers a likely assessment of extreme meteorological events' impact on human perceptivity, frame of mind or even health during driving which might have had a consequence as a car accident.

The area of the present research is focused on the region of Wielkopolska, located in the mid-western area of Poland. It is the second largest province in the country in terms of area and third in terms of population, and with one of the highest accident rates per 100,000 inhabitants. Research embraced an analysis of 26,641 car accidents during period 2010–2019 combined with four indices of extreme events like maximum daily temperature, maximum value of humidex, difference between maximum temperatures observed from day to day, and also difference between mean atmospheric pressure at the sea level observed from day to day. Spatial distribution of such indices is quite similar for stations at the regional scale, opposite to the wind speed or precipitation, which impact is rather more local. A distributed lag nonlinear model (DLNM) approach was used with Poisson regression models to obtain the relationship between extreme meteorological indices and car accidents. This study investigates the association between three indices previously insufficiently presented in the literature concerning car accidents. The results of this study can have important benefits for public health, especially with the accelerating and the progressive warming of climate.

In the following section, information on the Wielkopolska province is given, as well as the data used in the study are described more precisely. Further, findings of the analysis are presented where it is shown that an increase in indices results in an increase in the number of car accidents. This part is followed by discussion and conclusions.

## Materials and methods

This section summarizes the data and methods used for the work presented in this article. First the Wielkopolska region is characterized, then data on car accidents from the Polish Road Traffic Safety Observatory and meteorological data together with indices are discussed. Moreover, an information about the distributed lag non-linear model (DLNM) used in research is presented.

### Wielkopolska region

**Fig. 1:**
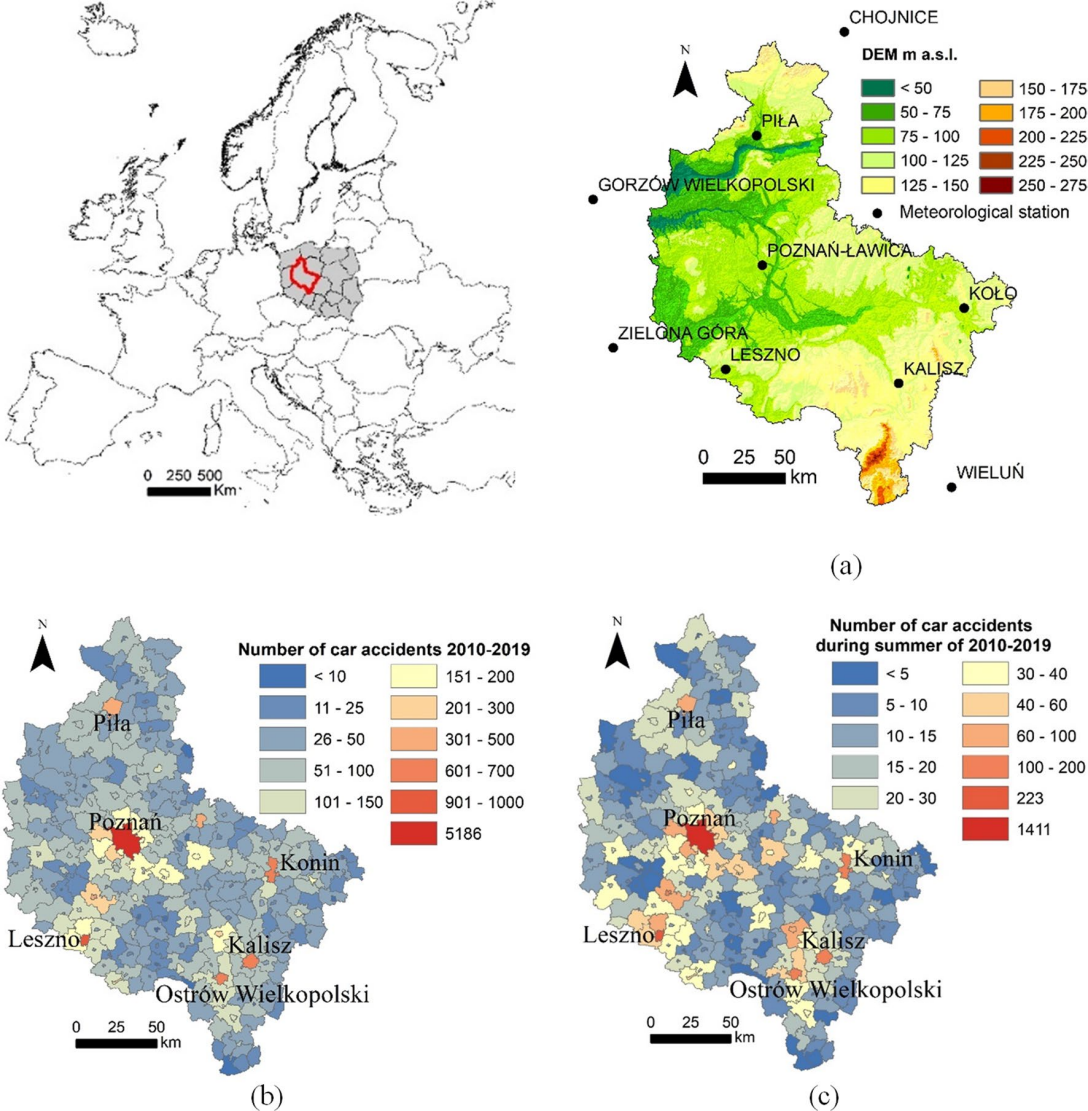
The Wielkopolska region of Poland: **a** Digital elevation model (DEM) with the location of meteorological stations; **b** Number of car accidents during period 2010–2019 for administrative units and towns with the highest number of car accidents during research period (above 400 car accidents); **c** Number of car accidents during summer period 2010–2019

The study focuses on the Wielkopolska province, located in the mid-western area of Poland. It is mostly lowland, much of the area lies below 150 m above sea level (a.s.l.), except for a part in the south that rises up to 275 m a.s.l. (Fig. 1a). Wielkopolska is a part of the Central European Lowland. The province covers 29,827 km^2^, which is 9.5% of the whole country of Poland. The region is inhabited by 3.496 million people. The Central Statistical Office (Statistics Poland 2022) reports that 53.7% of the region’s population lives in cities, more than half of which 52% in towns with more than 50,000 inhabitants, Poznań is the most populated one, with 570,000 inhabitants. The urbanization rate is lower than the national average of 59.9%.

According to Eurostat data on the number of cars per thousand inhabitants the analyzed region has one of the highest records in the country (Eurostat 2022a). In 2020, there were 721 vehicles per thousand inhabitants in Wielkopolska. Only in Opolskie and the capital region of Warsaw the numbers were higher: 727 and 750 respectively. One has to have in mind, that the average for Poland was 664 in 2020. What is also worth mentioning is the fact that in the last 20 years the number of cars per thousand inhabitants has more than doubled (313 in 2000). The number for Wielkopolska is also among the highest in the whole European Union (Eurostat 2022b). In case of the number of road accidents victims, Poland has the fourth highest record in Europe, with 66 victims in road accidents per million inhabitants (Eurostat 2022c). This number for the analyzed Wielkopolska region is below the country’s average and is 62, although it was 80 in a year earlier.

### Car accidents database

The database on car accidents was obtained from the Polish Road Traffic Safety Observatory. Records incorrectly recorded and accidents with the participation of people under the influence of prohibited substances, such as alcohol, drugs and other stimulants, have been removed from the database. In consequence 26,641 car accidents during period 2010–2019 have been analyzed. The number of analyzed car accidents in individual administrative units is presented in Fig. 1b and in Fig. 1c for summer period (i.e., June, July, August; 7482 car accidents). In this research, all traffic accidents have been included regardless of who was the perpetrator (car driver, pedestrian, cyclist) or what were the consequences of such an event.

### Distributed lag nonlinear model

A distributed lag non-linear model (DLNM) approach was used in this study with the package “*dlnm*” within the statistical environment R (Gasparrini 2011). The relationship between extreme meteorological indices and car accidents was modeled on a natural cubic B-spline with two equally spaced knots. The lag–response was modeled with considering a lag duration of 0–1 days. Month and day of the week were also included in the model. DLNM predictions are computed versus a reference value for the interpretation of the estimated effects. This can be a default value dependent on the function used for modeling the exposure-response, or can be set manually. In this research reference values were set to the values based on descriptive statistics as box plots presenting car accidents in relation to values of indices.

### Meteorological data and indices of extreme meteorological events

Hourly meteorological data, which are more representative than data with daily resolutions, have been obtained for nine stations from the Institute of Meteorology and Water Management (IMGW-PIB). Figure 1a presents meteorological stations used in this study located in and outside (but close to) the analyzed area. Four indices of extreme meteorological events were calculated to obtain a likely assessment of extreme meteorological events impact on human perceptivity, frame of mind or even health during driving which might have had a consequence as a car accident:

- Maximum daily temperature.
- Maximum value of humidex (presented in degree of Celsius, however, this value is without unit) based on equation introduced by Masterton and Richardson (1979). In case of high temperature increase, human body strives to maintain balance and reduce temperature through sweating. A high humidity influences heat exchange: the rate of evaporation decreases and less heat is lost from the skin’s surface. This results in discomfort of excessive heat. Humidex is a parameter expresses how the combined effects of warm temperatures and humidity are perceived. The value from 20 to 29 means comfortable conditions; above 30 to 39 means discomfort; from 40 to 45 almost everyone feels uncomfortable, and above 46 many types of labor must be restricted. For example, temperature of 32°C and humidity of 75% give a value of humidex of 46.2°C, and further increase of temperature poses a very serious threat to life. However, the heat balance mechanisms tend to decrease in effectiveness with age. It is calculated according to formula:
  $$H=T+h$$

where *H* is the humidex, *T* is the dry bulb temperature (°C), *h* is the 5/9 (*e*-10); *e* is the vapor pressure (mb or kPa*10).

Humidex was calculated in R package *ThermIndex*;

- Difference between maximum temperature calculated day to day (the absolute value);
- Difference between daily mean air pressure calculated day to day (the absolute value).

Maximum daily temperature for summer months from June to August was estimated based on hours’ data for every station, and the mean value for nine stations was calculated. In DLNM approach, reference values is setting as comfortable. In mortality research due to high temperatures it can be 21°C (Gasparrini 2011). In literature reference value is set depending on the analyzed issue, for example in research embracing mortality risk attributable to high and low ambient temperature it was temperature which corresponded to a minimum mortality percentile between the 1st and the 99th percentiles of temperature (Gasparrini et al. 2015). Similarly, in a study of Basagaña and de la Peña-Ramirez (2023) conducted for cold and hot temperatures may have impact in increase in risk of crashes, reference was set at the temperature of minimum risk of accidents between the 1st and 99th percentile of temperature.

In this study, the reference for maximum temperature (and also for humidex) was set to the value of minimum number of car accidents between 10th and 90th percentile of summer temperatures (also for humidex). Among summer temperatures during period 2010–2019, it was between 18.5°C and 29.7°C (between 19.5 and 33.8°C for humidex).

Based on hourly data for all year, maximum daily value of temperature was estimated, and then the differences between maximum temperature from day to day for nine stations and then, the mean value for these stations were calculated. Also based on hourly data for all year, mean daily value of atmospheric air pressure at the sea level was estimated, and then, the differences from day to day for seven stations (data not available for: Koło and Wieluń) were obtained, and the mean value for stations was calculated. The reference values for indices were obtained based on box plots presenting car accidents in relation to indices. The lag duration of 0–1 days was set for three indices, except the differences between air pressure.

## Results

### Statistics of car accidents in Wielkopolska region

**Fig. 2:**
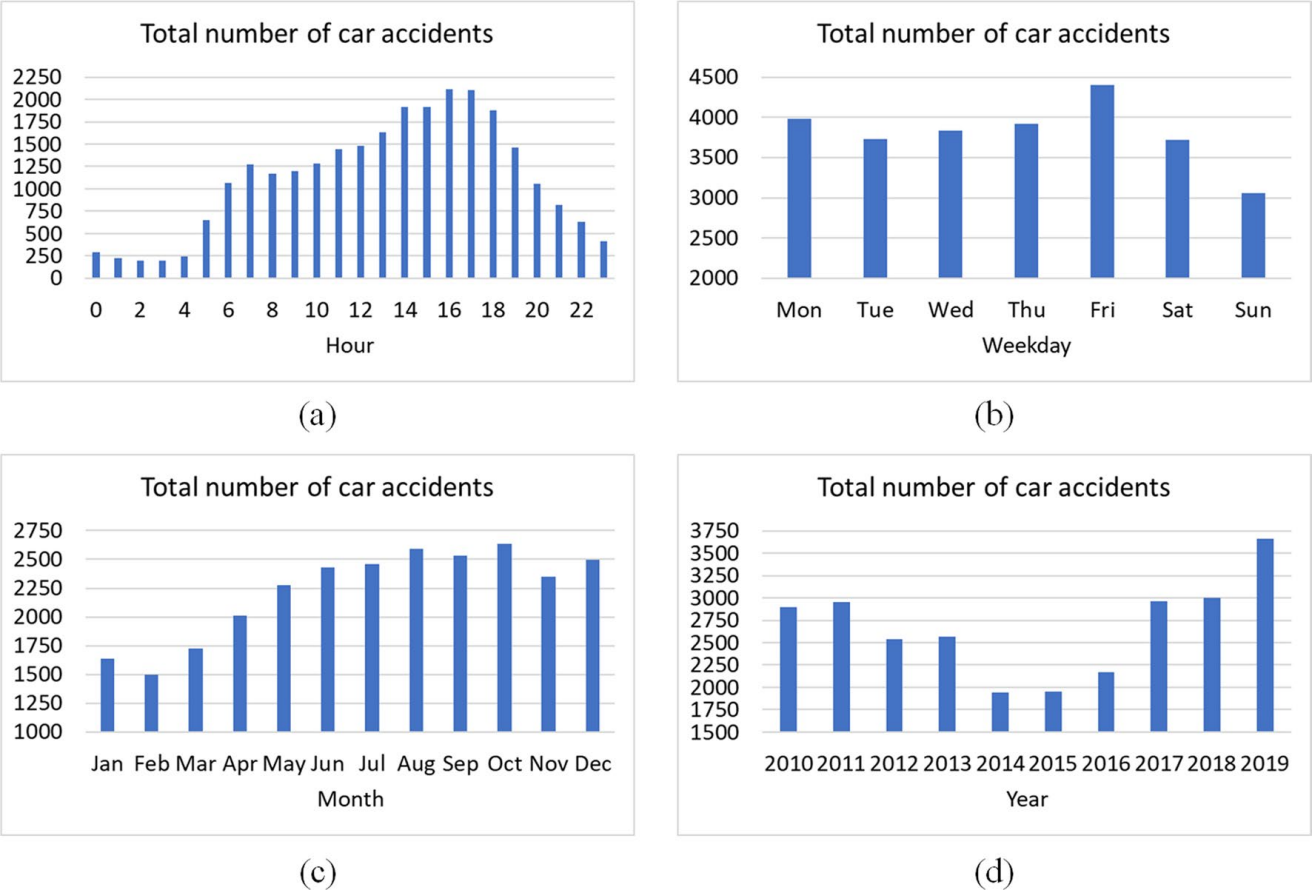
Total number of car accidents in Wielkopolska region during period 2010–2019 divided into: (**a**) hours; (**b**) weekdays; (**c**) months; (**d**) years

The smallest number of accidents (total value for the whole researched period below 1000 during every hour; see Table 1, Fig. 2a) took place during night hours from 9 pm to 5 am with the minimum at 2 am (196 events). Together with the intensifying daily traffic after 6 am, the number of accidents increases (above 1000 per every hour) and reaches its maximum at the time of afternoon traffic around 4 pm (2111) and 5 pm (2105). Daily the highest number (23) of such events occurred twice, both in 2011 on the 30th of September and 16th of December. The minimum yearly monthly sum was observed in March 2014: 111 accidents. During eleven months, the number of accidents exceeded value of 300; in this, the six highest were noted in 2019: in June, July, December, September, October, and August (monthly record: 387). According to the Traffic Bureau of the Police Headquarters (2011-2023), the number of car accidents after year 2011 in Poland decreases. However, during the years covered by this research 2010–2019 in Wielkopolska region, the smallest number of events took place in 2014 and 2015 (both years below 2000 accidents), but then, the trend was increasing with the highest number in 2019: 3662. After that year, in subsequent years from 2020 to 2022 in Wielkopolska, as in the whole country, there were less car accidents reordered (Traffic Bureau of the Central Police Headquarters 2011-2023). Moreover, the distribution of car accidents in Wielkopolska during these years is slightly different than for the whole Poland. During first 3 months of the year, the number of accidents for each month was below 2000. Then, the number of events was increasing and from June to December the value was high, from 2346 (in November), above 2400 during June and July, 2590 in August (summer holiday return period) to 2638 in October, what could be caused by worsening of visibility after dark or it may by impact of slippery surface caused by falling leaves. Total number of car accidents in Wielkopolska region during research period divided into hours, weekdays, months, and years is presented in Fig. 2. During summer, there were 7482 car events. In the case of car accidents broken down by road category, the highest number of accidents was recorded on county-level (poviat) roads (8852), then on municipal roads (6118) and also at the level of province roads: 5159.

**Table 1:** Statistics of car accidents in Wielkopolska region during period 2010−2019

Variable	Total number *N*	Hourly mean Mean ± SD	Hourly max
Hour			
0	287	1.06 ± 0.24	2
1	222	1.06 ± 0.28	3
2	196	1.03 ± 0.16	2
3	200	1.04 ± 0.20	2
4	246	1.04 ± 0.20	2
5	654	1.12 ± 0.35	3
6	1,061	1.21 ± 0.50	5
7	1,275	1.28 ± 0.61	5
8	1,166	1.22 ± 0.52	5
9	1,198	1.22 ± 0.49	4
10	1,280	1.21 ± 0.48	4
11	1,443	1.25 ± 0.53	5
12	1,479	1.22 ± 0.49	4
13	1,631	1.28 ± 0.59	5
14	1,917	1.34 ± 0.65	6
15	1,911	1.36 ± 0.66	6
16	2,111	1.39 ± 0.69	8
17	2,105	1.38 ± 0.70	6
18	1,874	1.33 ± 0.63	6
19	1,460	1.28 ± 0.55	5
20	1,056	1.17 ± 0.42	4
21	819	1.13 ± 0.38	4
22	633	1.11 ± 0.33	3
23	417	1.07 ± 0.27	3
Variable	Total number *N*	Daily mean Mean ± SD	Daily max
Weekday			
Mon	3,983	7.66 ± 3.71	22
Tue	3,731	7.18 ± 3.55	21
Wed	3,834	7.42 ± 3.76	21
Thu	3,913	7.60 ± 3.76	22
Fri	4,405	8.44 ± 4.25	23
Sat	3,720	7.18 ± 3.51	21
Sun	3,055	5.90 ± 3.10	18
Month			
Jan	1,639	5.41 ± 2.93	22
Feb	1,501	5.38 ± 2.96	19
Mar	1,723	5.61 ± 2.90	18
Apr	2,014	6.83 ± 3.28	18
May	2,274	7.38 ± 3.38	19
Jun	2,429	8.12 ± 3.69	21
Jul	2,463	7.95 ± 3.52	19
Aug	2,590	8.35 ± 3.54	21
Sep	2,531	8.44 ± 3.93	23
Oct	2,638	8.51 ± 3.93	22
Nov	2,346	7.82 ± 3.97	19
Dec	2,493	8.07 ± 4.36	23
Year			
2010	2,892	7.95 ± 3.71	21
2011	2,954	8.09 ± 3.70	23
2012	2,539	6.96 ± 3.33	22
2013	2,565	7.07 ± 3.43	18
2014	1,941	5.44 ± 2.98	21
2015	1,953	5.43 ± 2.60	15
2016	2,174	6.01 ± 2.97	18
2017	2,963	8.14 ± 3.94	22
2018	2,998	8.21 ± 3.77	21
2019	3,662	10.03 ± 4.11	22

### The reference values setting for DLNM approach

**Fig. 3:**
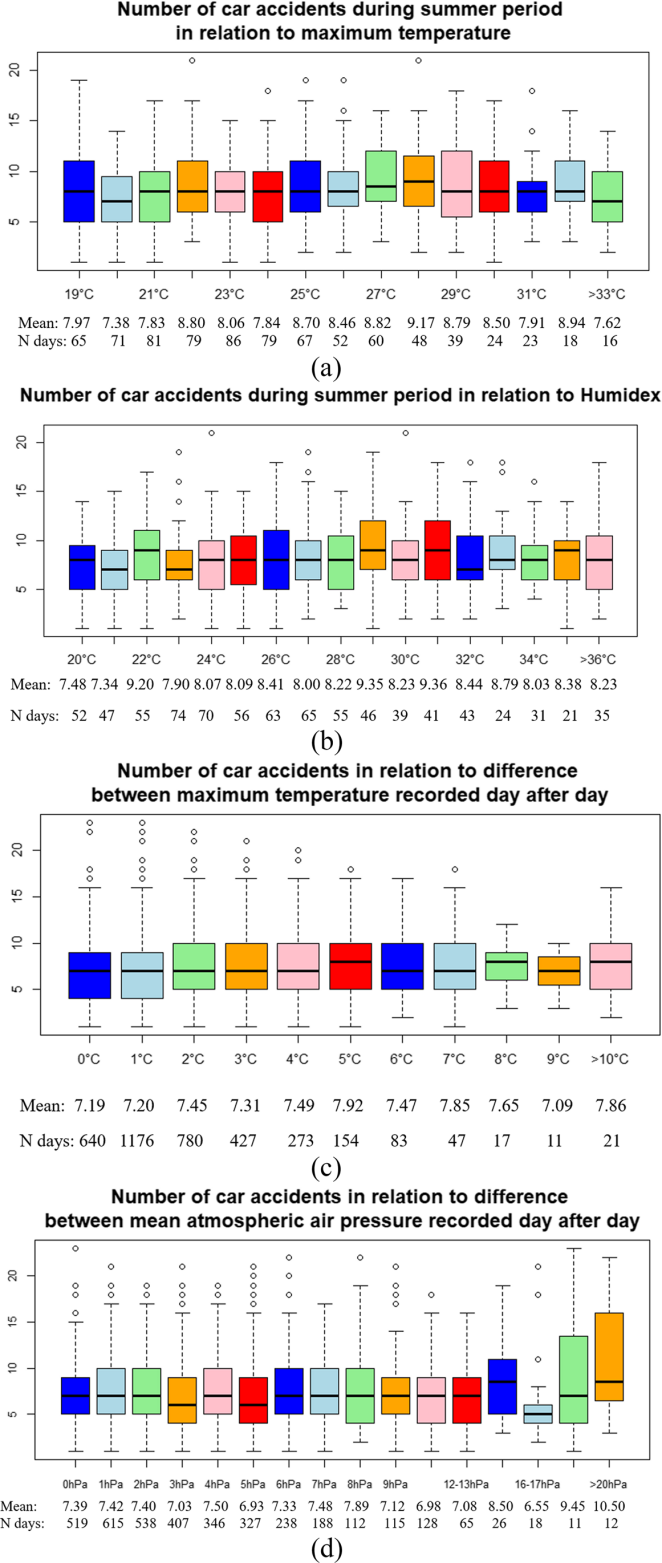
Box plots for number of car accidents during research period 2010–2019 in relation to indices: **a** maximum temperature during summer; **b** humidex during summer; **c** difference between maximum temperature recorded day after day; **d** difference between mean atmospheric air pressure recorded day after day. The mean value of car accidents and number of days with car accidents in particular values of indices are placed at the bottom

Reference values were obtained based on descriptive statistics presenting car accidents in relation to values of indices. This step of the study allowed to choose the optimum value. In the case of maximum temperature (Fig. 3a), the smallest value of car accident median, also the smallest mean number is observed at the temperature 20°C. It is 20th percentile of summer maximum temperature and 71st percentile of annual maximum temperature. For humidex, the relationships between number of car accidents and index is similar to maximum temperature, on which humidex is dependent. Also there the smallest values of median and mean is observed around the value of 20th percentile of summer humidex, i.e., 21.4°C (Fig. 3b). Through the lens of the analysis these temperatures also seems to be the most comfortable for human health and well-being.

For indices related to differences observed from day to day the choice of the reference value was more complicated, so the most reliable value seems to be the median: in the case of difference between maximum temperature recorded day after day, it was 2°C, where the number of car accidents was one of the smaller but not the smallest (Fig. 3c), and in the case of difference between mean atmospheric air pressure, it was 3.3 hPa, when the number of car accidents was one of the smallest (Fig. 3d).

### Impact of indices on traffic accidents

**Fig. 4:**
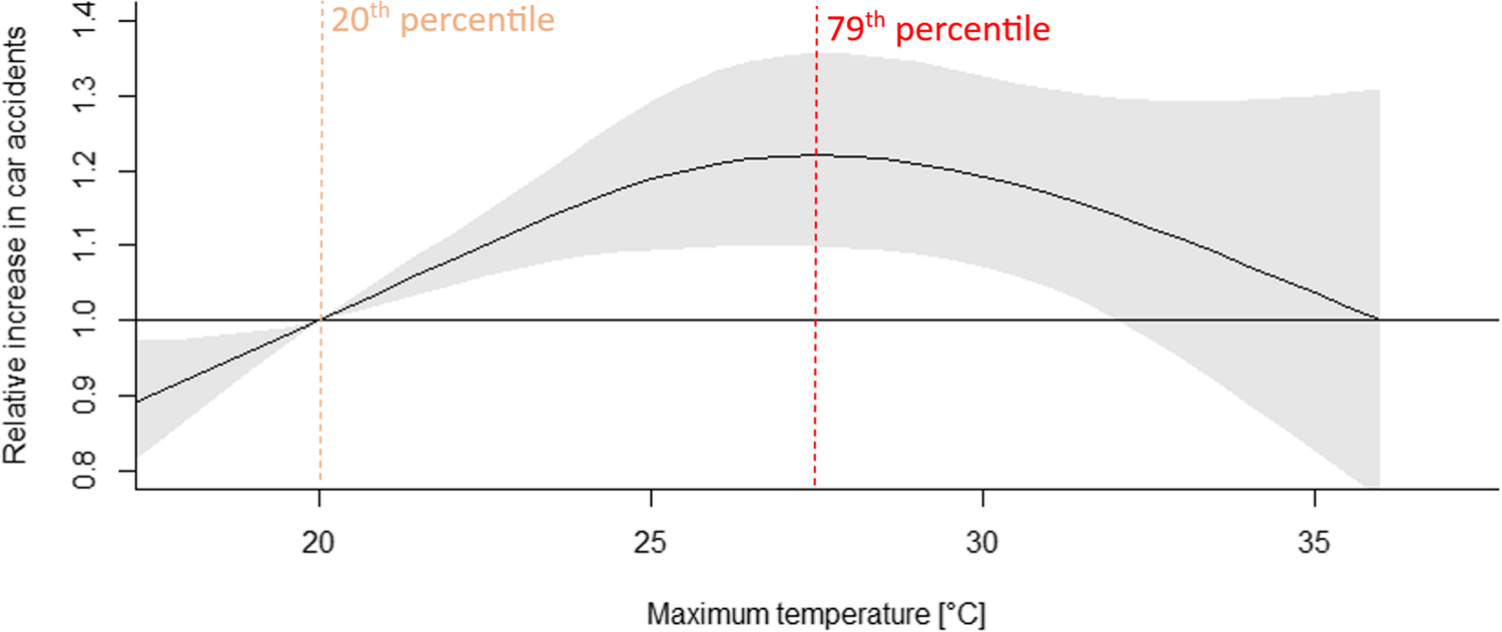
Relative increase in car accidents associated with maximum temperature (black solid line) with the 95% confidence interval CI (shaded gray). The vertical orange dashed line indicates the relative point and the red dashed line — maximum temperature representing a temperature value with the highest risk of car accidents, identified at the 79th percentile of the summer temperature or 27.5°C

**Fig. 5:**
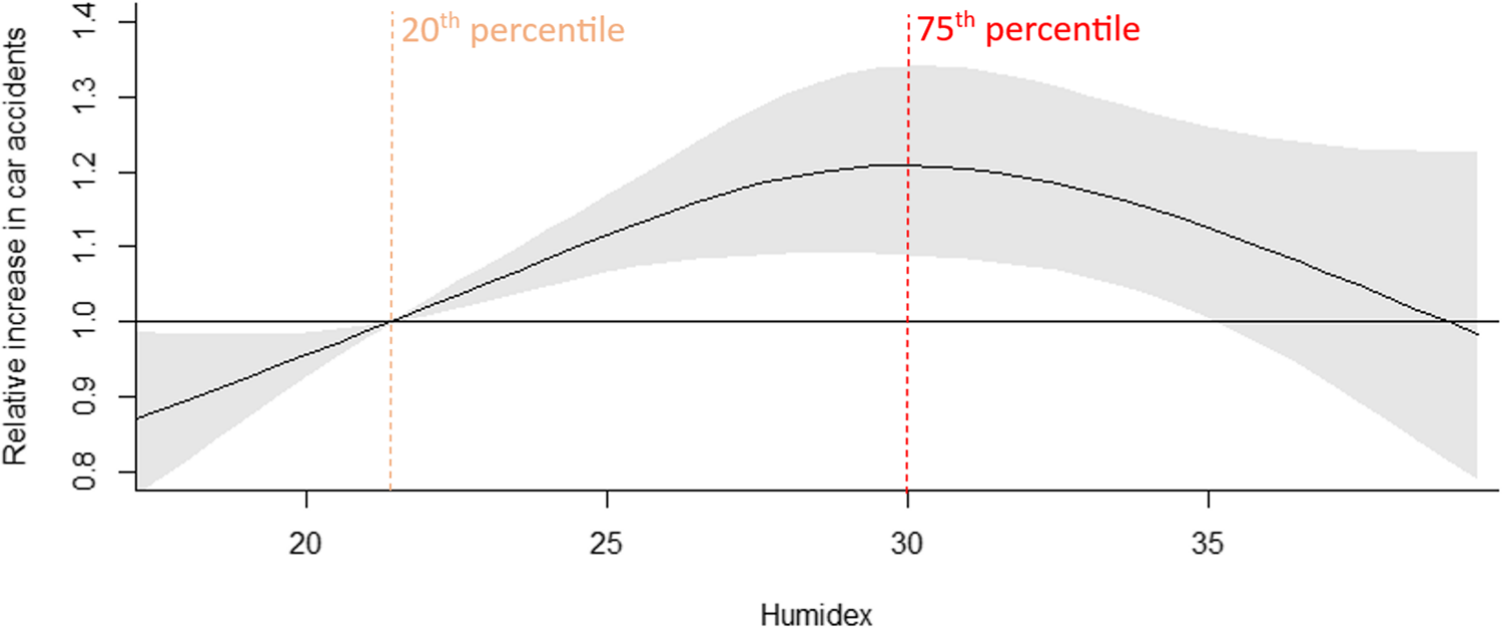
Relative increase in car accidents associated with Humidex (black solid line) with the 95% CI (shaded gray). The vertical orange dashed line indicates the relative point and the red dashed line — maximum humidex representing a value with the highest risk of car accidents, identified at the 75th percentile of the summer humidex or 30°C

**Fig. 6:**
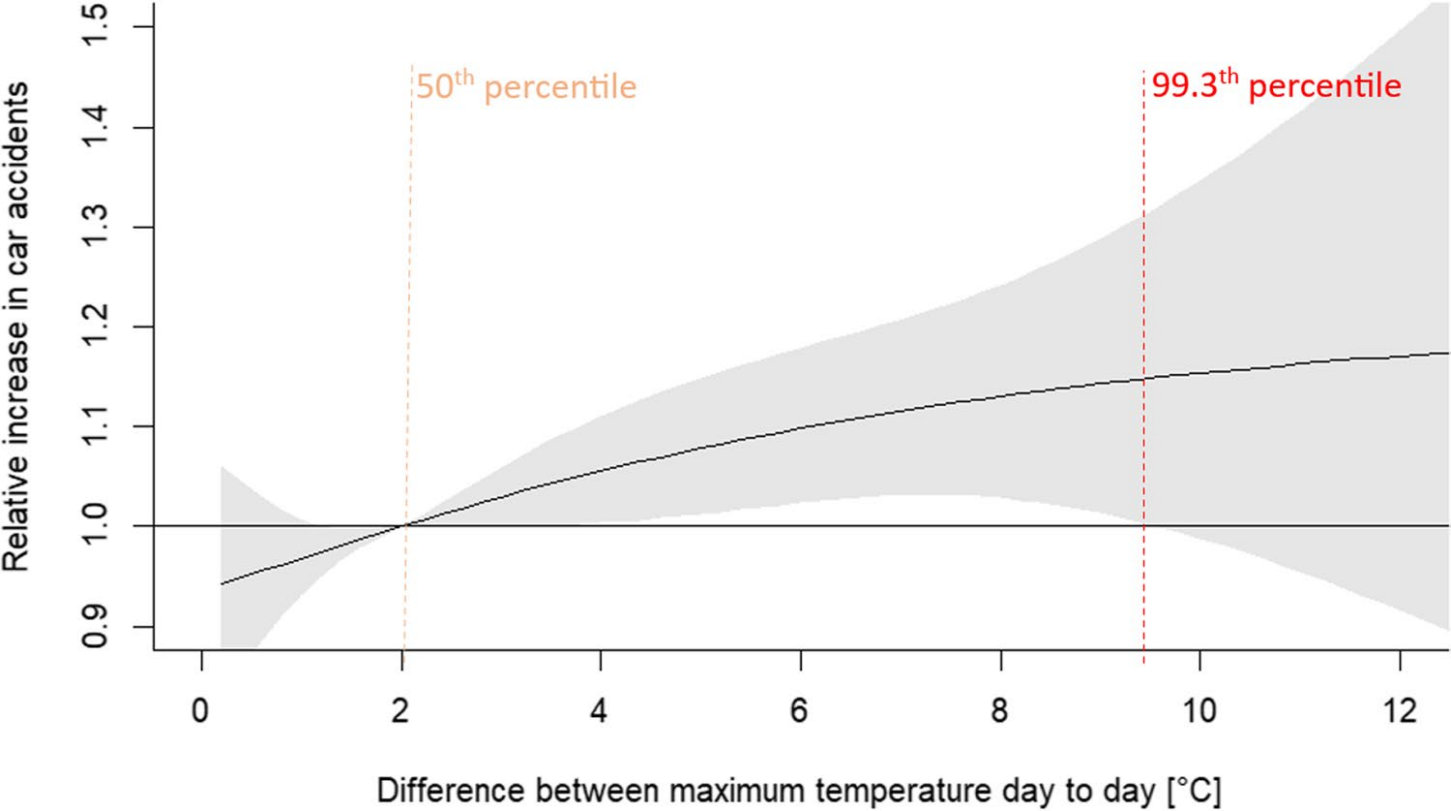
Relative increase in car accidents associated with difference between maximum temperature recorded day after day (black solid line) with the 95% CI (shaded gray). The vertical orange dashed line indicates the relative point and the red dashed line — difference between maximum temperature recorded day after day representing a value with the statistically significant highest risk of car accidents, identified at the 99.3th percentile of the difference or 9.5°C

**Fig. 7:**
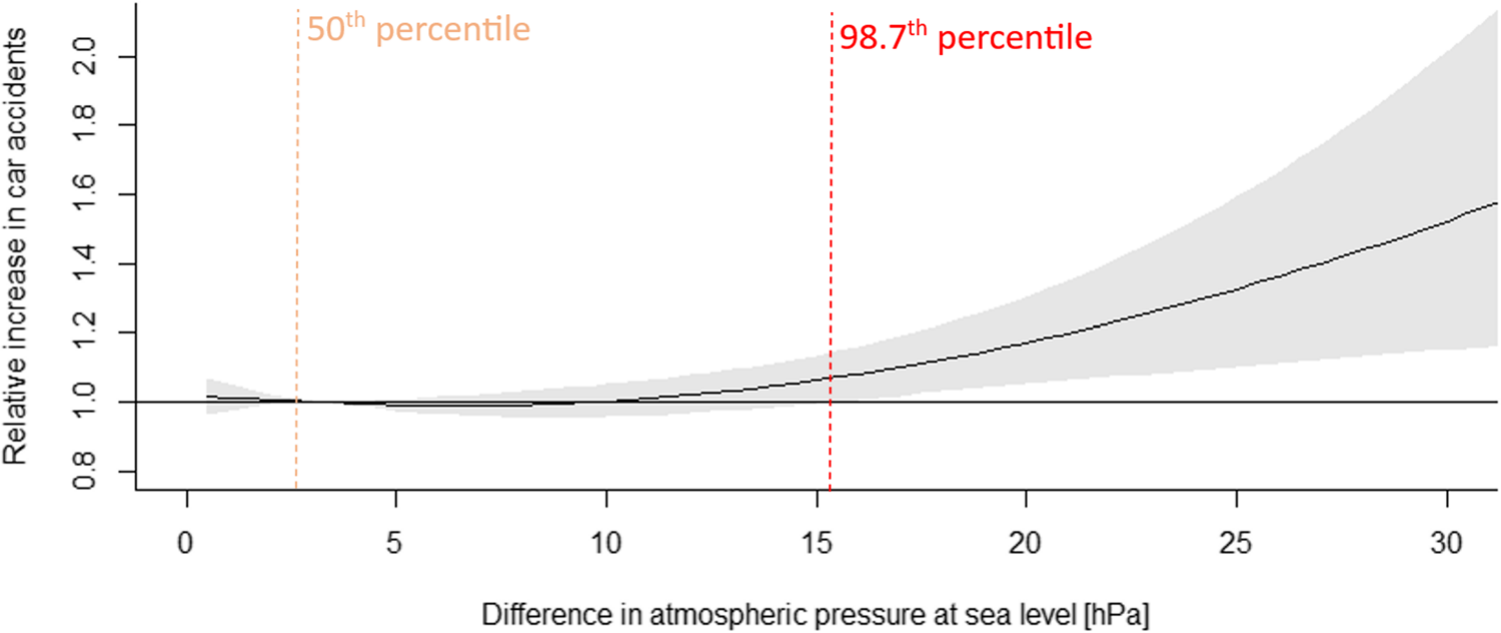
Relative increase in car accidents associated with difference between mean atmospheric air pressure recorded day after day (black solid line) with the 95% CI (shaded gray). The vertical orange dashed line indicates the relative point (50th percentile: 3.3 hPa) and the red dashed line — difference between maximum temperature recorded day after day representing a first value with the statistically significant risk of car accidents, identified at the 98.7th percentile of the difference or 15.5 hPa

This section presents results of four indices — traffic accidents association across Wielkopolska region. The exposure-response is computed versus a reference value for the interpretation of the estimated effects. In general, a higher value of index was associated with an increased risk of traffic accidents. However, in the case of two indices, the risk decreased after crossing the threshold. In Figs. 4, 5, 6, and 7, the vertical dashed lines were added to better presentations and understanding of results, similarly as was presented in Kim et al. (2019).

#### Impact of maximum temperature

Higher maximum air temperature is associated with an increased risk of car accidents. One can observe a nonlinear association (inverted J-shaped curve) with the highest risk at 27.5°C (79th percentile of summer value and 94th of annual value). The relative increase (RI) was 1.22 (95% CI: 1.30, 1.36) compared with the risk at the twentieth percentile of summer value (20°C) (Fig. 4, Table 2). By 32°C (96.4th percentile of summer and 99th of annual set of maximum temperature) is the last statistically significant result: 1.14 (95% CI: 1.00, 1.30).

**Table 2:** The relative increase in the number of car accidents as a response to the higher value of indices of extreme weather

Index	Value	RI (95% CI)
Maximum temperature for summer months June–August	Maximum: 27.5°C (79th percentile and annual 94th)	1.22 (1.10, 1.36)
	Last significant: 32°C (96.4th percentile and annual 99th)	1.14 (1.00, 1.30)
Humidex	30°C (75th percentile and annual 93rd)	1.21 (1.09, 1.34)
	Last significant: 35°C (94th percentile and annual 98.4th)	1.13 (1.01, 1.26)
Difference between maximum temperature day to day	9.5°C (99.3th percentile)	1.15 (1.00, 1.32)
Difference between atmospheric air pressure day to day	Statistically significant between:	1.07 (1.00, 1.15)
	15.5 hPa (98.7th percentile) and 19 hPa (99.5th percentile)	1.15 (1.04, 1.26)

#### Impact of humidex

Higher humidex is associated with an increased risk of car accidents. Here also is a nonlinear association (inverted J-shaped curve) with the highest risk at 30°C (75th percentile of summer value and 93rd of annual value). The relative increase (RI) was 1.21 (95% CI: 1.09, 1.34) compared with the risk at the twentieth percentile of summer value (21.4°C) (Fig. 5, Table 2). The last statistically significant value is noted by 35°C (94th percentile of summer and 98.4th of annual humidex). The relative increase by this was 1.13 (95% CI: 1.01, 1.26).

#### Impact of difference between maximum temperature day to day

The higher the difference between maximum temperature from day to day, the higher risk of car accidents. However, the highest value of difference, which is statistically significant, is up to 9.5°C. This difference corresponds to 99.3th percentile and relative increase by this is 1.15 (95% CI: 1.00, 1.32) (Fig. 6, Table 2).

#### Impact of the difference between mean air pressure at the sea level from day to day

Similar shape of the curve to the difference between maximum temperature from day to day has the higher difference between mean atmospheric air pressure calculated from day to day on a risk of car accidents. However, here all the highest value from 98.7th percentile of difference has a statistically significant impact. This value corresponds to a difference of 15.5 hPa. 99.5th percentile, it is difference of 19 hPa, could be considered as the most credible value and relative increase by this is 1.15 (95% CI: 1.04, 1.26) (Fig. 7, Table 2).

Table 2 summarizes the results. The highest risk, 1.22 and 1.21, respectively, is associated with occurrences of high maximum temperature and index linking maximum temperature and relative humidity. Thresholds with the highest risks have been estimated at 27.5°C for maximum temperature and 30°C for humidex. These records are not high in the context of heat waves, but as McLaren et al. (2005) reported, even at relatively cool ambient temperatures, the temperature rise in cars is significant on clear, sunny days. Additionally, in high temperatures people generally travel less and probably this has impact on fewer accidents overall. Analysis of car accidents in relation to values of maximum temperature revealed that above 33°C in all research period there were 16 days (Fig. 3a) and above humidex of 36°C there were 35 days (Fig. 3b). In one hand are less accidents and other hand such extreme temperatures are rare.

In the case of highest differences between maximum temperature from day to day and between mean atmospheric air pressure calculated from day to day, the increase in risk of car accidents is smaller: 1.15. The value of 19 hPa of difference between atmospheric air pressure was set as the highest statistically significant which can be considered credible.

**Fig. 8:**
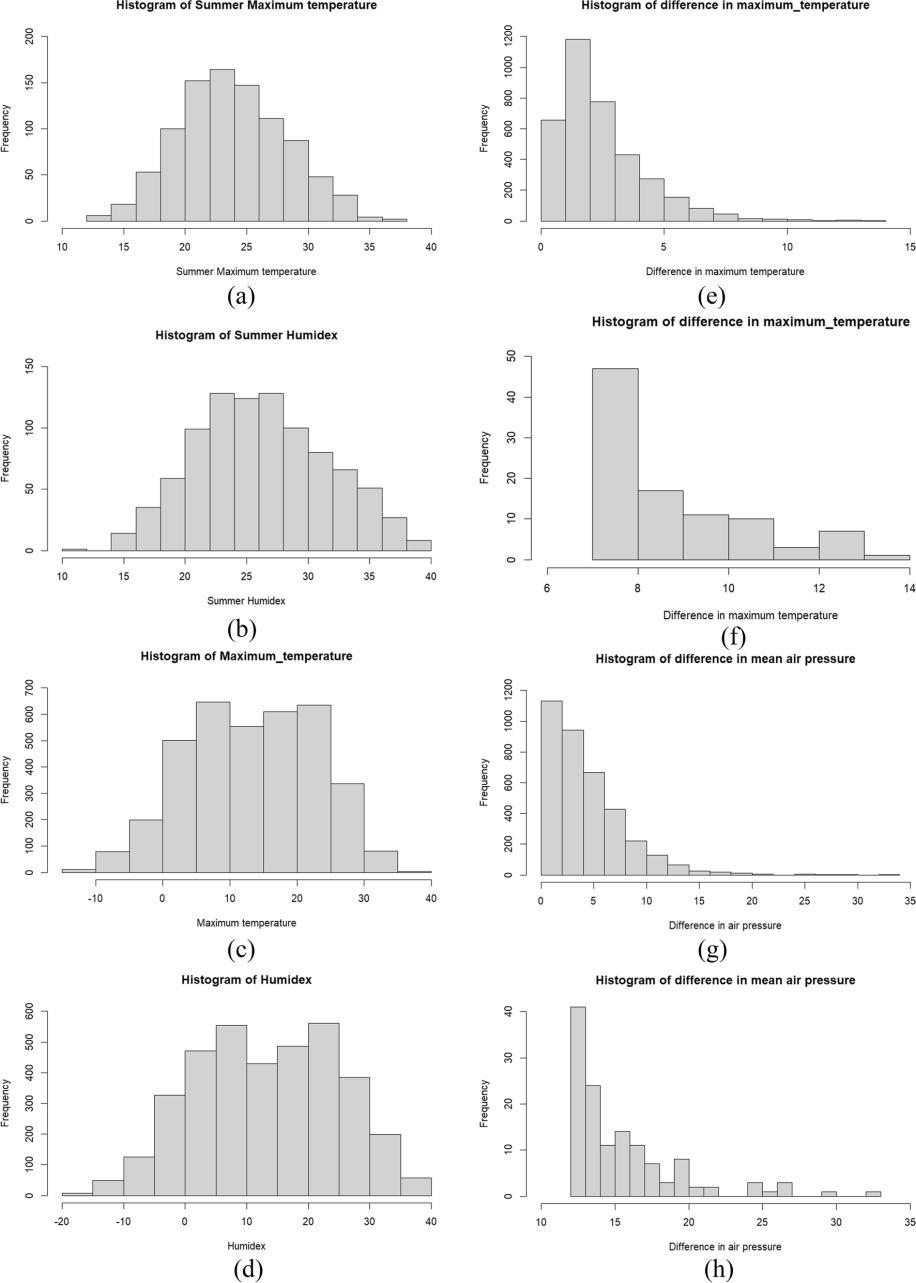
Histograms of all four indices. For Maximum temperature and humidex distribution was presented for summer (**a** and **b**) and for year (**c** and **d**, respectively); in the case of indices related to differences also are presented the highest extreme values (**e** and **f**: for difference in maximum temperature; **g** and **h** for difference in air pressure)

Figure 8 presents histograms of all indices. The distributions among them show that relatively high values of maximum temperature and humidex occurrence more often than high values of indices related to differences.

## Discussion

This study estimated the effects of indices based on data on accidents within the same climatic region of Wielkopolska with similar elevation. Month and day of the week were also taken into account. Car accidents that could have been caused by various types of illegal substances and medicines were excluded from the data. Results showed that higher values of extreme weather indices were associated with an increased risk of car accidents based on regional data. In the case of indices related to high temperature, i.e., maximum temperature and humidex, the association between them and car accidents showed nonlinearity (inverted J-shaped curve), with the risk of accidents is no longer increasing with index above some threshold. Based on research of the maximum temperature and humidex, it was possible to identify the critical values by which the risks of car accidents were the highest. It is possible that higher temperatures are felt more by road users, and they take action to reduce the possibility of an accident, such as reducing speed or avoiding leaving the house at high temperatures, especially in the case of elderly people.

For another two indices, i.e., difference between maximum temperature and difference between atmospheric air pressure calculated day to day, the association to risk in car accidents was more linear (J-shaped curve). However, the uncertainty is growing, when difference between maximum temperature will cross the threshold of very high 99.3th percentile. For the difference between atmospheric air pressure calculated day to day, the results are still significant to the highest percentiles, but also 99.5th percentile was set as the last significant.

In the research concerning the high temperature and suicide conducted by Kim et al. (2019), authors suggest that on the difference’s shapes of curves for the association (i.e., linear in cooler areas vs. nonlinear in warmer areas) may impact the larger number of occurrences of high temperatures in warmer sites, what allowed more precise estimates for the risk of suicide at extremely high temperature. Also, in this study the larger number of occurrences of indices of high temperatures and humidex compared with the smaller number of occurrences of high differences between maximum temperature from day to day and high differences between atmospheric air pressure calculated day to day during research period, could has impact on similar results in the difference's shapes of curves (i.e., inverted J-shaped curves for maximum temperature and humidex and nearly linear for indices related to differences from day to day). The fact that relationships between temperature and car accidents and humidex — car accidents was conducted for summer months, where the car, motor, bicycle and pedestrian traffic is the highest, may also impact findings in this research.

The results also reveal that road traffic participants are more sensitive to exposure to indices related to high temperature than to other indices in terms of the relationships between extreme weather indices and car accidents. This is especially interesting in the context of the more and more common availability of air conditioning in cars, which should mitigate the effects of high temperatures. Unfortunately, data on air conditioning in cars are not available, but one can suppose that in oldest car, which there is still a large number on Polish roads, the air cooling devices are not installed, or are malfunctioning. In a study for Spain (Basagaña and de la Peña-Ramirez 2023), analysis impact of cold and hot temperatures showed that the risk of crashes increased with temperature by 15% at the 99th percentile (reference was set at the temperature of minimum risk of accidents within the interval between the 1st and 99th percentile of temperature) and even higher when analyses were restricted to crashes with driver performance due to high temperature (23%). The authors of this research stated that cumulative fatigue caused by warm nights and difficulties in sleeping or cumulative dehydration leads to lower performance and using air conditioning in cars may not prevent against the increased risk of crashes during heat waves. Furthermore, in present study, the majority of traffic accidents happen in areas of towns, where there are numerous traffic jams. In such situations the temperatures inside the cars increase more than during smooth rides. Moreover, opening the windows does not bring a cooling effect. The results of Ding et al. (2022) showed that open windows have impact of 6.7 °C decrease in cabin air temperature where ambient temperature and solar radiation are high.

Elderly people are more vulnerable to the impact of extreme temperatures or changes in weather conditions, and it seems that they can cause more accidents. Wu et al. (2018) presented a significant positive association between fatal traffic crashes and heat waves with a 8.2% increase for 56–65 years old drivers. A study conducted by Park et al. (2021) showed that during hot temperatures elderly caused crashes between hours: 10:00–14:00 and 18:00–22:00; meanwhile, the increase in traffic accidents caused by young people was statistically significant at all times. Compared to the young, elderly drivers are more aware that their driving skills are not at their best, and they adapt to the conditions by decreasing the speed of their cars or avoid dangerous situations on the roads; however, under hot or changing weather conditions, they may still have trouble with detecting and reacting to specific hazards (Bromberg et al. 2012). Young drivers are often unaware how their dangerous behavior (e.g., night driving or risky driving behaviors, e.g., peer passengers, speeding) may have drastic consequences on the roads (Curry et al. 2015). He at al. 2023 in global study of road injuries due to high temperature stressed that males aged 25–49 are more susceptible on influence of high temperature. Wu et al. (2023) also pointed out that male drivers’ cognitive efforts in high temperature were significantly higher than in medium-temperature environment.

In a research associated with the occurrence of trauma, car accidents among them, Abe et al. (2008) found significant association with high temperature, but no impact of barometric pressure and humidity.

The present study has some limitations. The not too high number of cases limits more accurate analysis and consideration of other factors. The analysis did not take into account the influence of precipitation. Their impact is much more local; nevertheless, to some extent, it could have a disturbing effect on the results. Calculations omitted conditions associated with precipitation also due to lack of hourly data (there are only 6h total available), fatal and nonfatal injuries, elderly and young drivers, as well as male and female drivers, also it does not account for speeding, distracted driving, and traffic volume on the road at the time of collision. However, as was mentioned above, every traffic participant may have be impacted by car accidents, as analyzed indices affect all groups of drivers and traffic participants. Additionally, in this study, values of differences of air pressure and maximum temperature were computed as absolute values without differentiation on positive or negative changes. It could have an impact in some cases. In a research about impact of change in temperature on depression and suicide conducted by Holopainen et al. (2014), authors gave a hypothesis stating that people from exposed groups are more vulnerable to an increase of temperature and are more tolerant to cold.

The total number of road accidents is probably much higher than the one included in the database. Due to financial matters (insurance reasons and high and often obligatory fines), minor road accidents (without health and large material consequences) very often are not reported.

## Conclusions

Higher values of the first group of indices (i.e., maximum air temperature and humidex) are associated with an increased risk of car accidents on the levels of 1.22 and 1.21, respectively. Based on inverted J-shaped curves, the highest value of indices with maximum risk in car accidents were estimated at the threshold of 27.5°C for maximum temperature and 30°C for humidex. The associations in the second group of high differences (between maximum temperature from day to day and between mean atmospheric air pressure calculated from day to day) on a risk of car accidents give smaller increase in car accident risk at the level of 1.15. The shapes of curves are also different in these two groups: in first there is inverted J-shaped with lower value of high percentiles indicated the maximum risk (79th and 75th, i.e. 94th and 93rd of annual value, respectively); among second group, the curves are more linear with the very high percentile (99.3th and 99.5th, respectively).

This study investigates the association between three indices previously insufficiently presented in the literature concerning car accidents. The results of this study can have important benefits for public health, especially with the accelerating and the progressive warming of climate and for region with one of the highest number of road accidents victims in Europe.

## Data Availability

The authors do not have permission to share data obtained from the Polish Road Traffic Safety Observatory.

## References

[CR1] Abe T, Tokuda Y, Ohde S, Ishimatsu S, Nakamura T, Birrer RB (2008). The influence of meteorological factors on the occurrence of trauma and motor vehicle collisions in Tokyo. Emerg Med J.

[CR2] Abohassan A, El-Basyouny K, Kwon TJ (2022). Effects of inclement weather events on road surface conditions and traffic safety: an event-based empirical analysis framework. Trans Res Rec.

[CR3] Agarwal M, Maze TH, Souleyrette R (2005) Impacts of weather on urban freeway traffic flow characteristics and facility capacity. In Proceedings of the 2005 Mid-continent Transportation Research Symposium (Vol 9). https://intrans.iastate.edu/app/uploads/2023/08/Proc_Midcon_2005.pdf. Accessed 25 May 2023

[CR4] Asher MI, García-Marcos L, Pearce NE, Strachan DP (2020). Trends in worldwide asthma prevalence. Europ Respir J.

[CR5] Bachmair S, Svensson C, Prosdocimi I, Hannaford J, Stahl K (2017). Developing drought impact functions for drought risk management. Nat Hazard Earth Syst Sci.

[CR6] Barriopedro D, Fischer EM, Luterbacher J, Trigo RM, García-Herrera R (2011). The hot summer of 2010: redrawing the temperature record map of Europe. Science.

[CR7] Basagaña X, de la Peña-Ramirez C (2023). Ambient temperature and risk of motor vehicle crashes: a countrywide analysis in Spain. Environ Res.

[CR8] Basagaña X, Escalera-Antezana JP, Dadvand P, Llatje Ò, Barrera-Gómez J, Cunillera J, Medina-Ramón M, Pérez K (2015). High ambient temperatures and risk of motor vehicle crashes in Catalonia, Spain (2000–2011): a time-series analysis. Environ Health Perspect.

[CR9] Bastos A, Ciais P, Friedlingstein P, Sitch S, Pongratz J, Fan L (2020). Direct and seasonal legacy effects of the 2018 heat wave and drought on European ecosystem productivity. Sci Adv.

[CR10] Billot R, El Faouzi NE, De Vuyst F (2009). Multilevel assessment of the impact of rain on drivers’ behavior: standardized methodology and empirical analysis. Trans Res Rec.

[CR11] Bromberg S, Oron-Gilad T, Ronen A, Borowsky A, Parmet Y (2012). The perception of pedestrians from the perspective of elderly experienced and experienced drivers. Accid Anal Prev.

[CR12] Choryński A, Matczak P, Jeran A, Witkowski M (2023). Extreme weather events and small municipalities’ resilience in Wielkopolska Province (Poland). Int J Disaster Risk Reduction.

[CR13] Christenson ML, Geiger SD, Anderson HA (2013). Heat-related fatalities in Wisconsin during the summer of 2012. WMJ.

[CR14] Curry AE, Pfeiffer MR, Durbin DR, Elliott MR (2015). Young driver crash rates by licensing age, driving experience, and license phase. Acc Anal Prev.

[CR15] Ding X, Zhang W, Yang Z, Wang J, Liu L, Gao D, Guo D, Xiong J (2022). Effect of open-window gaps on the thermal environment inside vehicles exposed to solar radiation. Energies.

[CR16] Dixon PG, Kalkstein AJ (2018). Where are weather-suicide associations valid? An examination of nine US counties with varying seasonality. Int J Biometeorol.

[CR17] Eisenberg D, Warner KE (2005). Effects of snowfalls on motor vehicle collisions, injuries, and fatalities. Amer J Pub Health.

[CR18] ETSC (2022) Ranking EU progress on road safety. 16th Road Safety Performance Index Report June 2022 https://etsc.eu/16th-annual-road-safety-performance-index-pin-report/, access data: 8 May 2023

[CR19] Eurostat (2022) Stock of vehicles by category and NUTS 2 regions. https://ec.europa.eu/eurostat/databrowser/view/TRAN_R_VEHST__custom_6207028/default/table?lang=en. Accessed 8 May 2023 (in Polish)

[CR20] Eurostat (2022) Stock of vehicles by category and NUTS 2 regions. https://ec.europa.eu/eurostat/databrowser/view/TRAN_R_VEHST__custom_6320571/default/table?lang=en. Accessed 8 May 2023 (in Polish)

[CR21] Eurostat (2022) Victims in road accidents by NUTS 2 regions. https://ec.europa.eu/eurostat/databrowser/view/TRAN_R_ACCI__custom_6321723/default/table?lang=en. Accessed 8 May 2023 (in Polish)

[CR22] Gabriel KM, Endlicher WR (2011). Urban and rural mortality rates during heat waves in Berlin and Brandenburg, Germany. Environ Pollut.

[CR23] Gasparrini A (2011). Distributed lag linear and non-linear models in R: the package dlnm. J Stat Softw.

[CR24] Gasparrini A, Guo Y, Hashizume M, Lavigne E, Zanobetti A, Schwartz J, Tobias A, Tong S, Rocklöv J, Forsberg B, Leone M, De Sario M, Bell ML, Guo YL, Wu CF, Kan H, Yi SM, de Sousa Zanotti Stagliorio Coelho M, Saldiva PH, Honda Y, Kim H, Armstrong B. (2015) Mortality risk attributable to high and low ambient temperature: a multicountry observational study. Lancet. 386(9991):369-75. 10.1016/S0140-6736(14)62114-0 10.1016/S0140-6736(14)62114-0 PMC4521077 26003380

[CR25] Gill M, Goldacre MJ (2009). Seasonal variation in hospital admission for road traffic injuries in England: analysis of hospital statistics. Injury Prevention.

[CR26] Graczyk D, Kundzewicz ZW, Choryński A, Førland EJ, Pińskwar I, Szwed M (2019). Heat-related mortality during hot summers in Polish cities. Theor Appl Climatol.

[CR27] Graczyk D, Pińskwar I, Choryński A (2022). Heat-related mortality in two regions of poland: focus on urban and rural areas during the most severe and long-lasting heatwaves. Atmosphere.

[CR28] Gu L, Chen J, Yin J, Sullivan SC, Wang HM, Guo S, Zhang L, Kim JS (2020). Projected increases in magnitude and socioeconomic exposure of global droughts in 1.5 and 2° C warmer climates. Hydrol Earth Syst Sci.

[CR29] He L, Liu C, Shan X, Zhang L, Zheng L, Yu Y, Tian X, Xue B, Zhang Y, Qin X, Wang C, Zhang K, Luo B (2023). Impact of high temperature on road injury mortality in a changing climate, 1990–2019: a global analysis. Sci Total Environ.

[CR30] Hiltunen L, Ruuhela R, Ostamo A, Lönnqvist J, Suominen K, Partonen T (2012). Atmospheric pressure and suicide attempts in Helsinki, Finland. Int J Biometeorol.

[CR31] Hjelkrem OA, Ryeng EO (2016). Chosen risk level during car-following in adverse weather conditions. Accid Anal Prev.

[CR32] Holopainen J, Helama S, Partonen T (2014). Does diurnal temperature range influence seasonal suicide mortality? Assessment of daily data of the Helsinki metropolitan area from 1973 to 2010. Int J Biometeorol.

[CR33] Jaroszweski D, McNamara T (2014). The influence of rainfall on road accidents in urban areas: a weather radar approach. Travel Behav Soc.

[CR34] Keay K, Simmonds I (2005). The association of rainfall and other weather variables with road traffic volume in Melbourne, Australia. Accid Anal Prev.

[CR35] Kenney WL, Craighead DH, Alexander LM (2014) Heat waves, aging, and human cardiovascular health. Med Sci Sports Exerc 46(10):1891 10.1249/MSS.0000000000000325 PMC4155032 24598696

[CR36] Kim JK, Ulfarsson GF, Shankar VN, Mannering FL (2010). A note on modeling pedestrian-injury severity in motor-vehicle crashes with the mixed logit model. Acc Anal Prev.

[CR37] Kim Y, Kim H, Gasparrini A, Armstrong B, Honda Y, Chung Y (2019). Suicide and ambient temperature: a multi-country multi-city study. Environ Health Perspect.

[CR38] Kron W, Eichner J, Kundzewicz ZW (2019). Reduction of flood risk in Europe–reflections from a reinsurance perspective. J Hydrol.

[CR39] Kuchcik M (2021). Mortality and thermal environment (UTCI) in Poland—long-term, multi-city study. Int J Biometeorol.

[CR40] Kundzewicz ZW, Huang J, Pińskwar I, Su B, Szwed M, Jiang T (2020). Climate variability and floods in China-a review. Earth-Sci Rev.

[CR41] Leard B, Roth K (2015) Weather, traffic accidents, and climate change (No. RFF DP 15-19). Resources for the Future. https://media.rff.org/archive/files/sharepoint/WorkImages/Download/RFF-DP-15-19.pdf. Accessed 25 May 2023

[CR42] Liu A, Soneja SI, Jiang C, Huang C, Kerns T, Beck K (2017). Frequency of extreme weather events and increased risk of motor vehicle collision in Maryland. Sci Total Environ.

[CR43] Lu M, Yu Z, Hua J, Kang C, Lin Z (2023). Spatial dependence of floods shaped by extreme rainfall under the influence of urbanization. Sci Total Environ.

[CR44] Masterton JM, Richardson FA (1979) Humidex: a method of quantifying human discomfort due to excessive heat and humidity. Environ Can Atmos Environ. https://publications.gc.ca/collections/collection_2018/eccc/En57-23-1-79-eng.pdf. Accessed 25 May 2023

[CR45] McLaren C, Null J, Quinn J (2005). Heat stress from enclosed vehicles: moderate ambient temperatures cause significant temperature rise in enclosed vehicles. Pediatrics.

[CR46] McMichael AJ, Woodruff RE, Hales S (2006). Climate change and human health: present and future risks. Lancet.

[CR47] Mohamed MG, Saunier N, Miranda-Moreno LF, Ukkusuri SV (2013). A clustering regression approach: a comprehensive injury severity analysis of pedestrian–vehicle crashes in New York, US and Montreal, Canada. Safe Sci.

[CR48] Oke O, Dougherty E, Rasmussen KL, Morrison RR, Carter E (2023). Spatial distribution of socio-demographic and housing-based factors in relation to flash and slow-rise flooding hazards in the US. Environ Res Lett.

[CR49] Ombadi M, Risser MD (2022). What's the temperature tomorrow? Increasing trends in extreme volatility of daily maximum temperature in Central and Eastern United States (1950–2019). Weather Clim Extremes.

[CR50] Park J, Choi Y, Chae Y (2021). Heatwave impacts on traffic accidents by time-of-day and age of casualties in five urban areas in South Korea. Urban Climate.

[CR51] Pińskwar I, Choryński A, Kundzewicz ZW (2020). Severe drought in the Spring of 2020 in Poland—more of the same?. Agronomy.

[CR52] Robine JM, Cheung SL, Le Roy S, Van Oyen H, Herrmann FR (2007) Report on excess mortality in Europe during summer 2003. EU Community Action Programme for Public Health, https://ec.europa.eu/health/ph_projects/2005/action1/docs/action1_2005_a2_15_en.pdf, access data: 4 May 2023

[CR53] Rodrigues M, Santana P, Rocha A (2020). Modelling climate change impacts on attributable-related deaths and demographic changes in the largest metropolitan area in Portugal: a time-series analysis. Environ Res.

[CR54] Rodrigues M, Santana P, Rocha A (2021). Modelling of temperature-attributable mortality among the elderly in Lisbon metropolitan area, Portugal: a contribution to local strategy for effective prevention plans. J Urban Health.

[CR55] Semenza JC, McCullough JE, Flanders WD, McGeehin MA, Lumpkin JR (1999). Excess hospital admissions during the July 1995 heat wave in Chicago. Amer J Prevent Med.

[CR56] Seneviratne SI, Zhang X, Adnan M, Badi W, Dereczynski C, Di Luca A, Ghosh S, Iskandar I, Kossin J, Lewis S, Otto F, Pinto I, Satoh M, Vicente-Serrano SM, Wehner M, Zhou B (2021) Weather and climate extreme events in a changing climate. In: Climate Change 2021: The Physical Science Basis. Contribution of Working Group I to the Sixth Assessment Report of the Intergovernmental Panel on Climate Change. Masson-Delmotte V, et al. (eds.). Cambridge University Press, Cambridge, United Kingdom and New York, NY, USA, 1513–1766. 10.1017/9781009157896.013, https://www.ipcc.ch/report/ar6/wg1/chapter/chapter-11/. Accessed 25 May 2023

[CR57] Sieg T, Thieken AH (2022). Improving flood impact estimations. Environ Res Lett.

[CR58] Statistics of Police (2022) https://statystyka.policja.pl/st/przestepstwa-ogolem/przestepstwa-drogowe/prowadzenie-pojazdu-w-s/122332,Prowadzenie-pojazdu-w-stanie-nietrzezwosci.html. Accessed 25 May 2023 (in Polish)

[CR59] Statistics Poland, Statistical yearbook of the regions – Poland. Warsaw (2022) https://stat.gov.pl/obszary-tematyczne/roczniki-statystyczne/roczniki-statystyczne/rocznik-statystyczny-wojewodztw-2021,4,16.html, access data: 25 May 2023

[CR60] Su X, Zhi D, Song D, Tian L, Yang Y (2023). Exploring weather-related factors affecting the delay caused by traffic incidents: mitigating the negative effect of traffic incidents. Sci Total Environ.

[CR61] Traffic Bureau of the Central Police Headquarters (2011-2023). Reports from several years. https://dlakierowcow.policja.pl/dk/statystyka/100254,Wypadki-drogowe-raporty-roczne.html. Accessed 8 May 2023 (in Polish)

[CR62] Trombley J, Chalupka S, Anderko L (2017). Climate change and mental health. AJN Amer J Nurs.

[CR63] Urban A, Davídkovová H, Kyselý J (2014). Heat-and cold-stress effects on cardiovascular mortality and morbidity among urban and rural populations in the Czech Republic. Int J Biometeorol.

[CR64] Van Lanen HA, Laaha G, Kingston DG, Gauster T, Ionita M, Vidal JP (2016). Hydrology needed to manage droughts: the 2015 European case. Hydrol Process.

[CR65] Vos T, Lim SS, Abbafati C, Abbas KM, Abbasi M, Abbasifard M (2020). Global burden of 369 diseases and injuries in 204 countries and territories, 1990–2019: a systematic analysis for the Global Burden of Disease Study 2019. Lancet.

[CR66] Wang X, Lavigne E, Ouellette-kuntz H, Chen BE (2014). Acute impacts of extreme temperature exposure on emergency room admissions related to mental and behavior disorders in Toronto, Canada. J Affect Disord.

[CR67] WHO (2023) Asthma. https://www.who.int/news-room/fact-sheets/detail/asthma, access data: 8 May, 2023

[CR68] Wu CY, Zaitchik BF, Gohlke JM (2018). Heat waves and fatal traffic crashes in the continental United States. Accid Anal Prev.

[CR69] Wu Z, Jin T, Chen C, Liu X, Yan J (2023). How do different ambient temperatures and vehicle speeds affect the cognitive performance of male drivers? Evidence from ERP. Traffic Inj Prev.

[CR70] Yang B, Cui Q, Meng Y, Zhang Z, Hong Z, Hu F (2023). Combined multivariate drought index for drought assessment in China from 2003 to 2020. Agric Water Manag.

[CR71] Yin Q, Wang J (2017). The association between consecutive days’ heat wave and cardiovascular disease mortality in Beijing, China. BMC Public Health.

[CR72] Zhou X, Wang Q, Yang T (2020). Decreases in days with sudden day-to-day temperature change in the warming world. Global Planet Change.

[CR73] Ziska LH, Makra L, Harry SK, Bruffaerts N, Hendrickx M, Coates F (2019). Temperature-related changes in airborne allergenic pollen abundance and seasonality across the northern hemisphere: a retrospective data analysis. Lancet Planet Health.

